# Supplementary material for: Multi-omics analysis reveals the dynamic interplay between Vero host chromatin structure and function during vaccinia virus infection
Source: Commun Biol. 2024 Jun 11;7:721. doi: 10.1038/s42003-024-06389-x (PMC11166932; doi:10.1038/s42003-024-06389-x)
Supplement: Supplementary file 2 — Supplementary Information [file 42003_2024_6389_MOESM2_ESM.docx]

Supplementary Information

**Multi-omics analysis reveals the dynamic interplay between Vero host chromatin structure and function during vaccinia viral infection**

Vrinda Venu^1^, Cullen Roth^2^, Samantha H. Adikari^3^, Eric M. Small^1^, Shawn R. Starkenburg^2^, Karissa Y. Sanbonmatsu^4,5^, Christina R. Steadman^1^*

^1^Climate, Ecology & Environment Group, Los Alamos National Laboratory, Los Alamos, New Mexico, USA

^2^Genomics & Bioanalytics Group, Los Alamos National Laboratory, Los Alamos, New Mexico, USA

^3^Biochemistry & Biotechnology Group, Los Alamos National Laboratory, Los Alamos, New Mexico, USA

^4^Theoretical Biology and Biophysics Group, Los Alamos National Laboratory, Los Alamos, New Mexico, USA

^5^New Mexico Consortium, Los Alamos, New Mexico, USA

*corresponding author: [crsteadman@lanl.gov](mailto:crsteadman@lanl.gov)


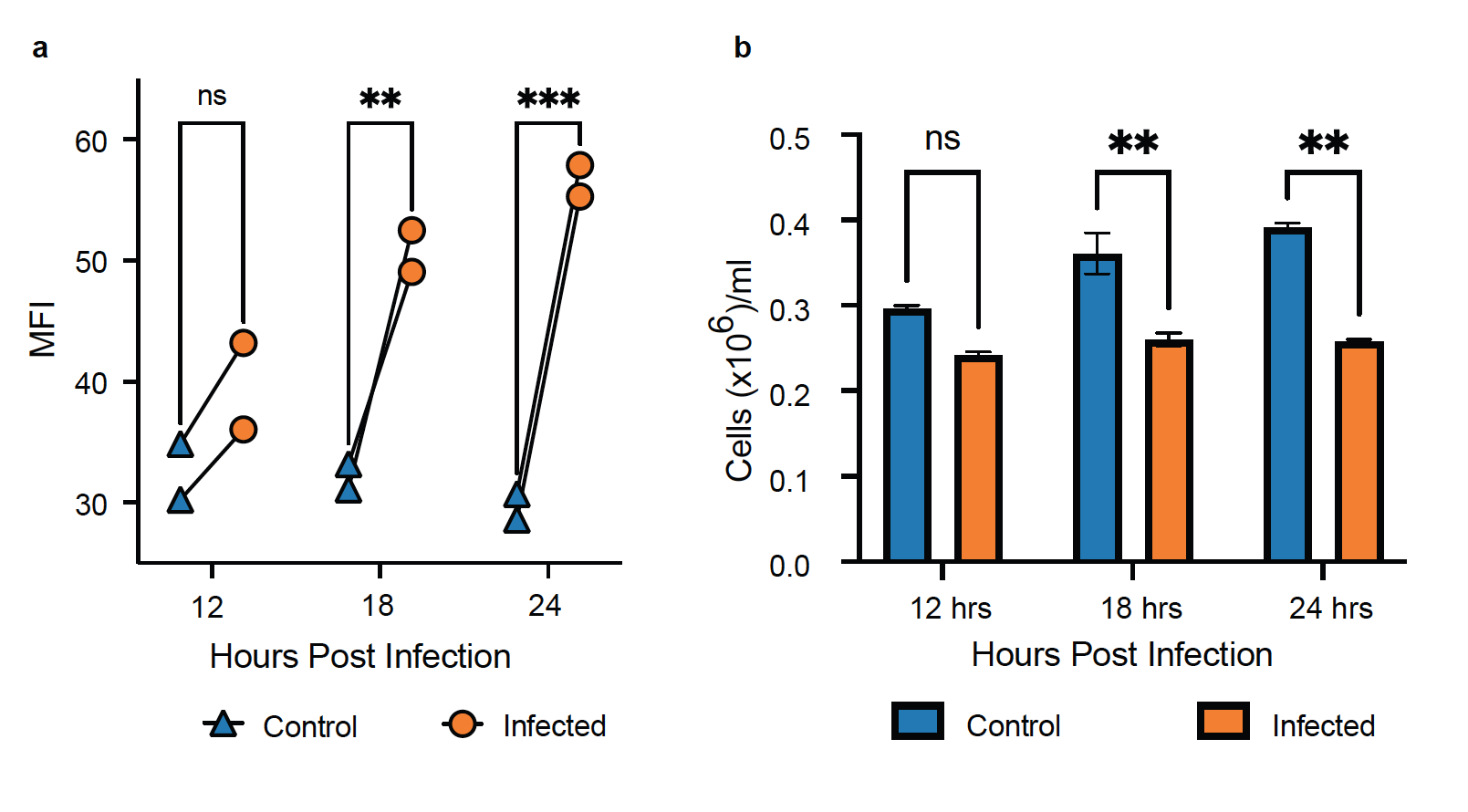


**Figure S1: a)** Infection efficiency was verified by immunofluorescence using an anti-vaccinia virus monoclonal antibody. Mean fluorescent intensity from mock-infected control and infected samples are plotted for all three time points. **b)** Cell viability in mock-infected control and infected samples are plotted for all three time points. n=3 biological replicates per condition, **p-value < .01


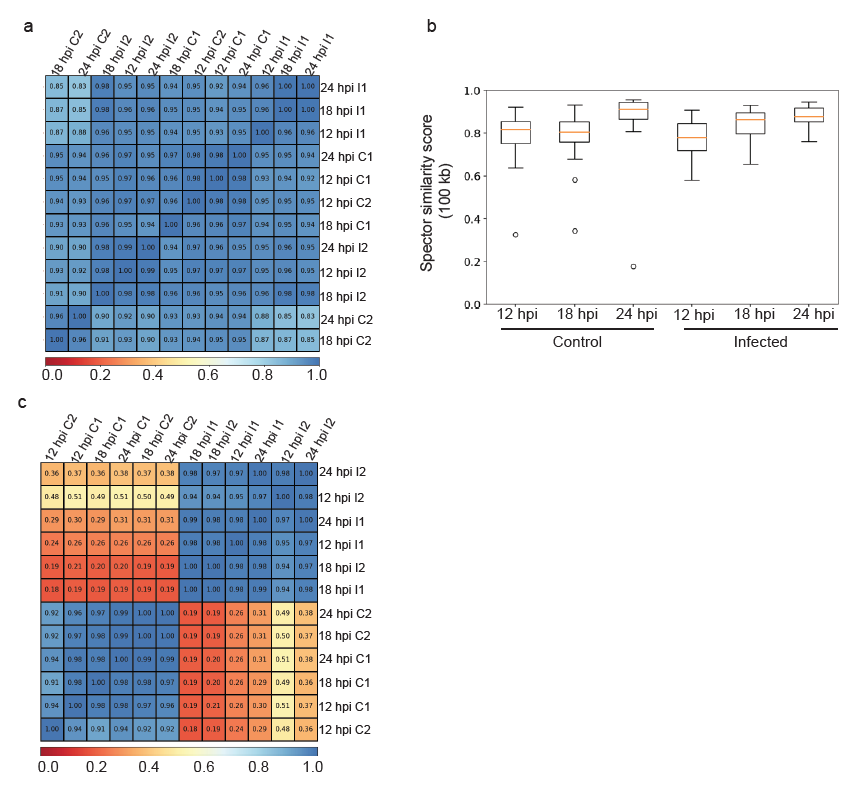


**Figure S2: a)** Genome-wide Pearson correlation between ATAC-seq samples at 10 kb resolution. **b)** Spector similarity score estimated at 100 kb resolution between biological replicates for each condition per time point. **c)** Genome-wide Pearson correlation between RNA-seq samples at 10 kb resolution.


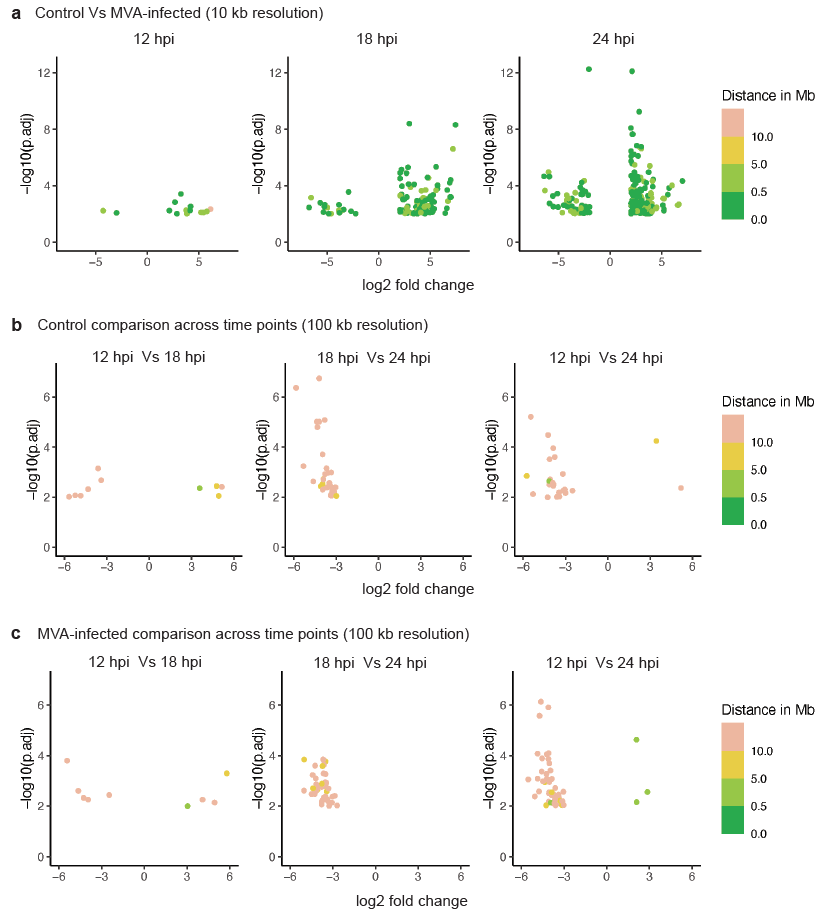


**Figure S3: a)** Genomic regions with significant differences in the number of contacts between mock-infected control and infected cells were identified using multiHiCcompare at 10 kb resolution and plotted as log2 fold change in contact frequency (x-axis) and negative log_10_ adjusted p-value (y-axis). Color scale annotates the distance between contacting regions. Points in the positive axis represent infection-bias regions (more contacts due to MVA infection than in control) and points in the negative axis represent control-bias regions. Genomic regions with significant differences in the number of contacts between time points, **b)** in control cells, and **c)** in MVA-infected cells were identified using multiHiCcompare at 100 kb resolution and plotted as log_2_ fold change in contact frequency (x-axis) and negative log_10_ adjusted p-value (y-axis). Color scale annotates the distance between contacting regions. Points in the positive axis represent regions with more contacts at a later time point and vice versa.


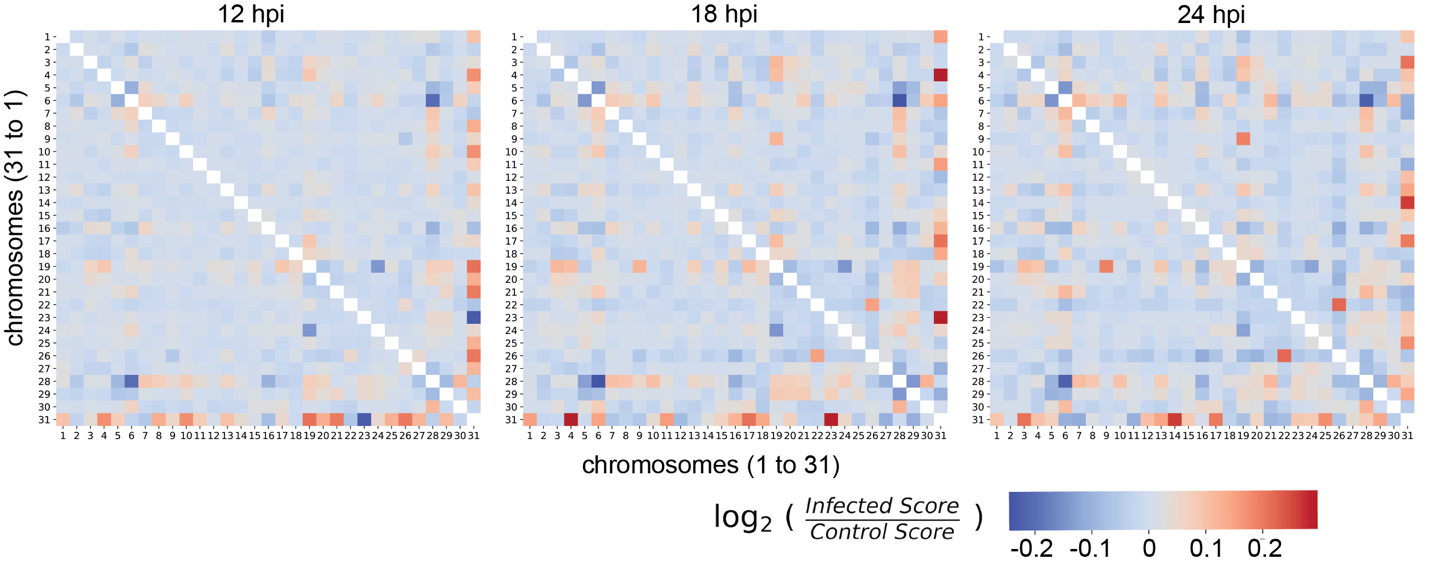


**Figure S4:** Heat map representing differential contacts between pairs of chromosomes at all three (12, 18, 24 hpi) timepoints. Color scale represents log_2_[infected score/control score]. Chromosome pairs that have increased contacts in MVA-infected cells are colored in shades of red, and pairs that have increased contacts in control cells are colored in shades of blue.


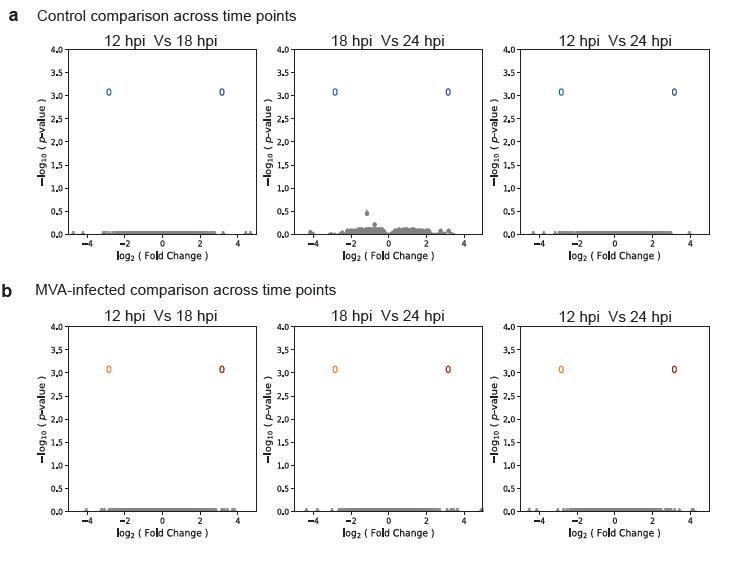


**Figure S5:** Volcano plots represent loop comparison between time points in **a)** mock-infected control and **b)** MVA-infected cells. Grey dots represent all called loops however, zero differential (adjusted p-value < 0.05) loops were identified in all pair wise comparisons.


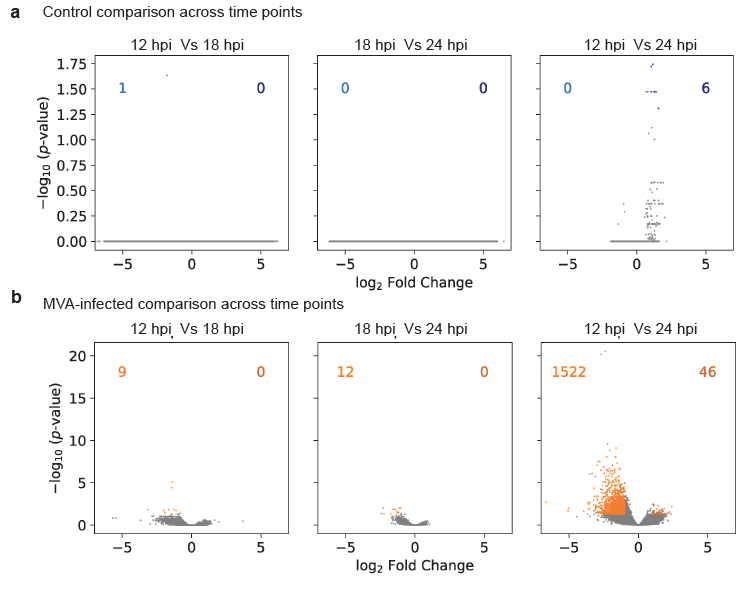
 **Figure S6:** Volcano plots demonstrate differentially accessible open chromatin regions (OCRs) between time points in **a)** mock-infected control and **b)** MVA-infected cells. Grey dots represent all OCRs. Significantly different (adjusted p value<0.05) OCRs with log_2_ fold change < -1 or >1 are colored. Points in the positive axis represent differential OCRs with accessibility at a later time point and vice versa.


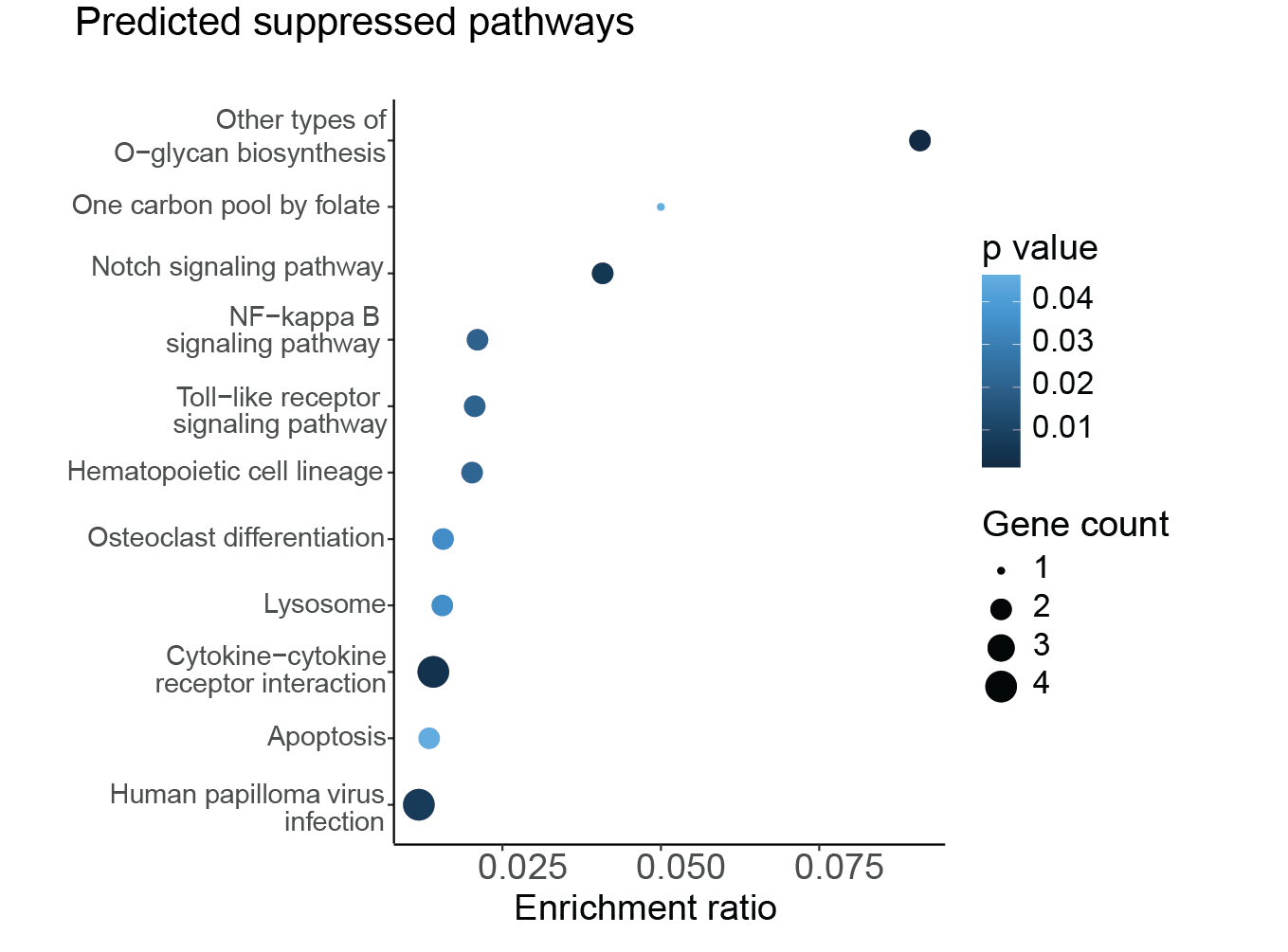


**Figure S7:** Gene regulatory pathways that are potentially suppressed due to MVA infection, as predicted by Kegg Orthology analysis of control-biased differentially accessible genes. Pathways are shown in the decreasing order of their gene enrichment ratio.


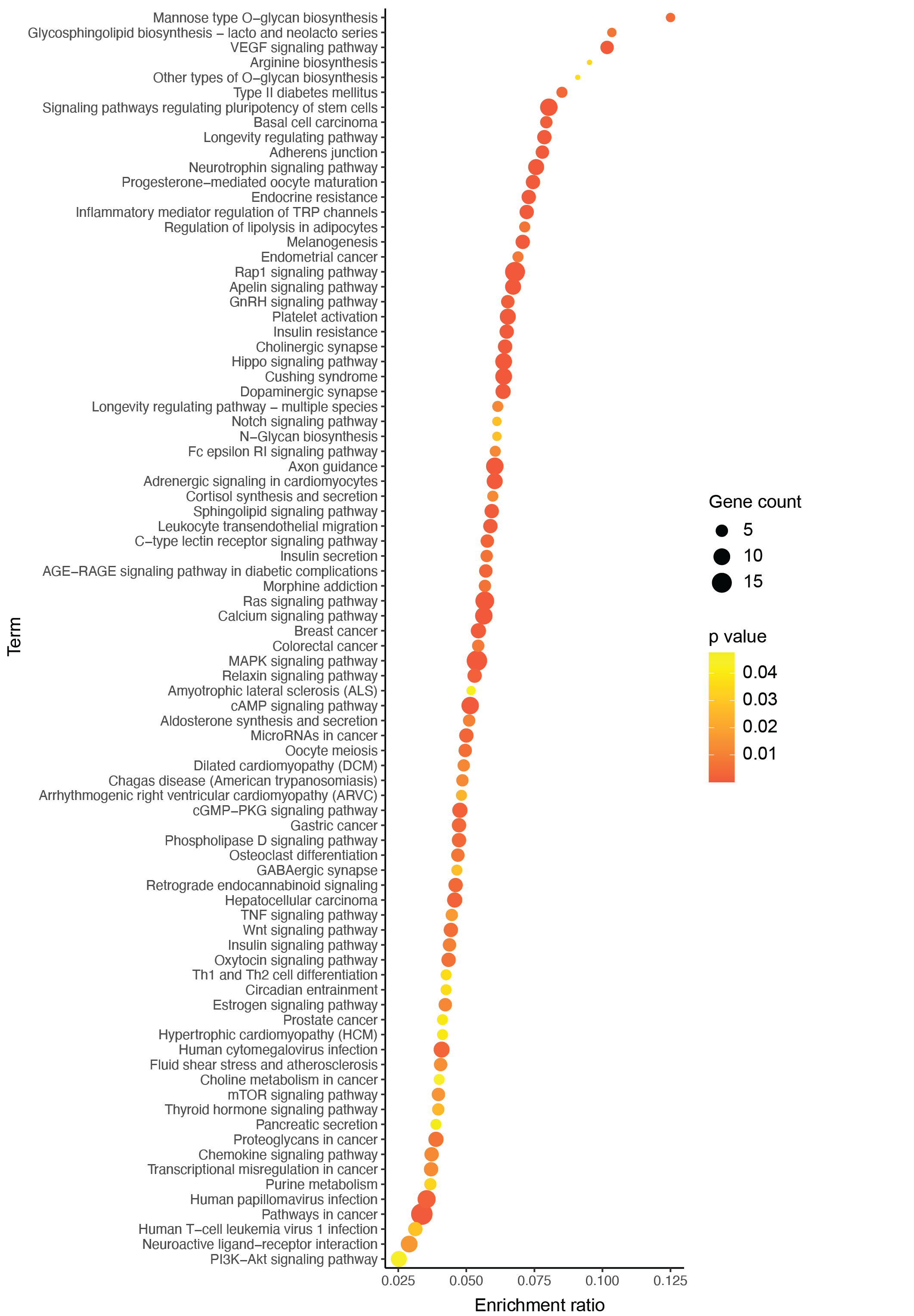


**Figure S8:** Gene regulatory pathways that are potentially activated due to MVA infection, as predicted by Kegg Orthology analysis of infection-biased differentially accessible genes. Pathways are shown in decreasing order of their gene enrichment ratio.


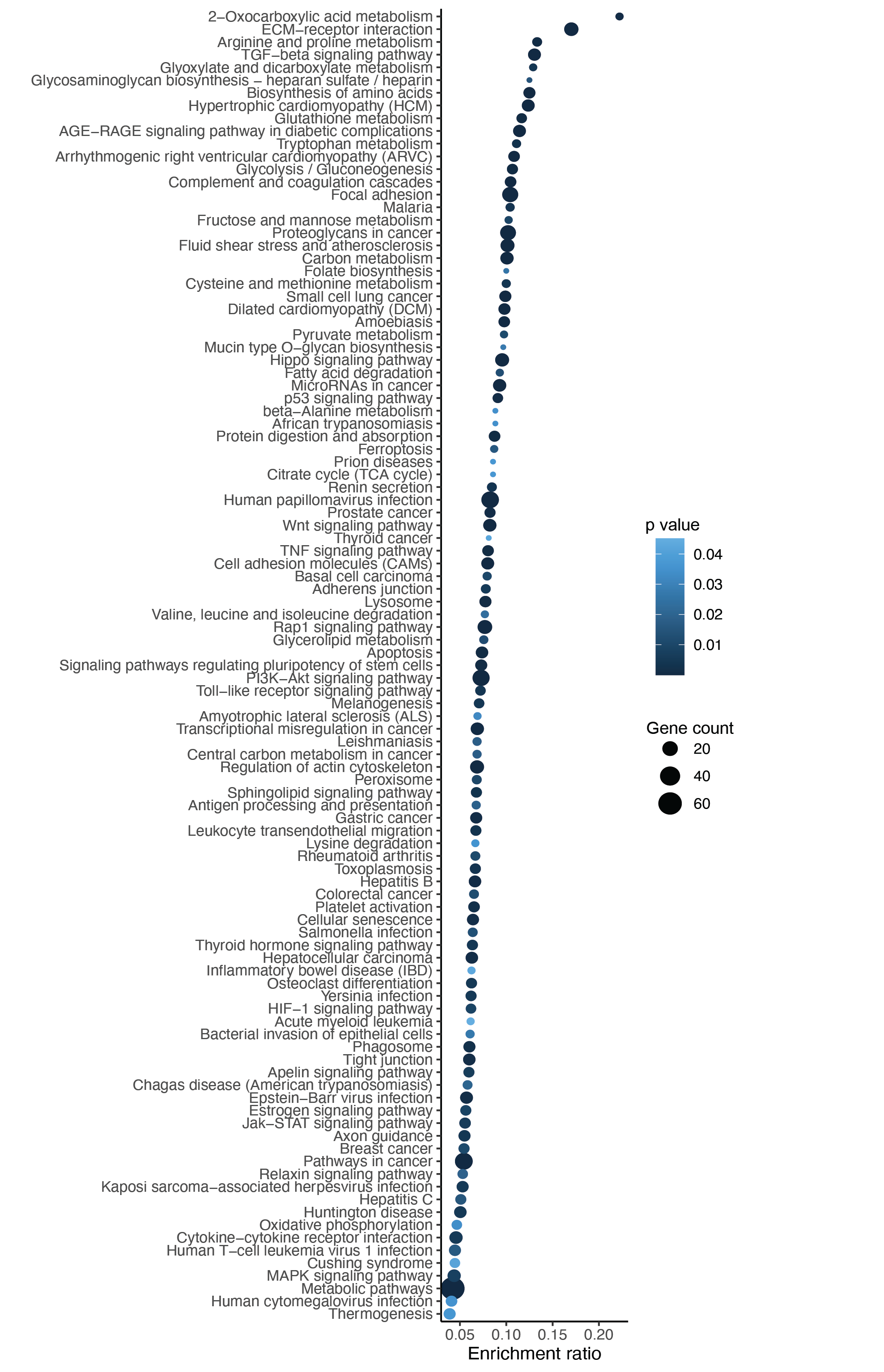


**Figure S9:** Gene regulatory pathways that are potentially suppressed due MVA infection, as identified by Kegg Orthology analysis of downregulated genes. Pathways are shown in the decreasing order of their gene enrichment ratio. (Extended list of main text Figure 6a, left panel).


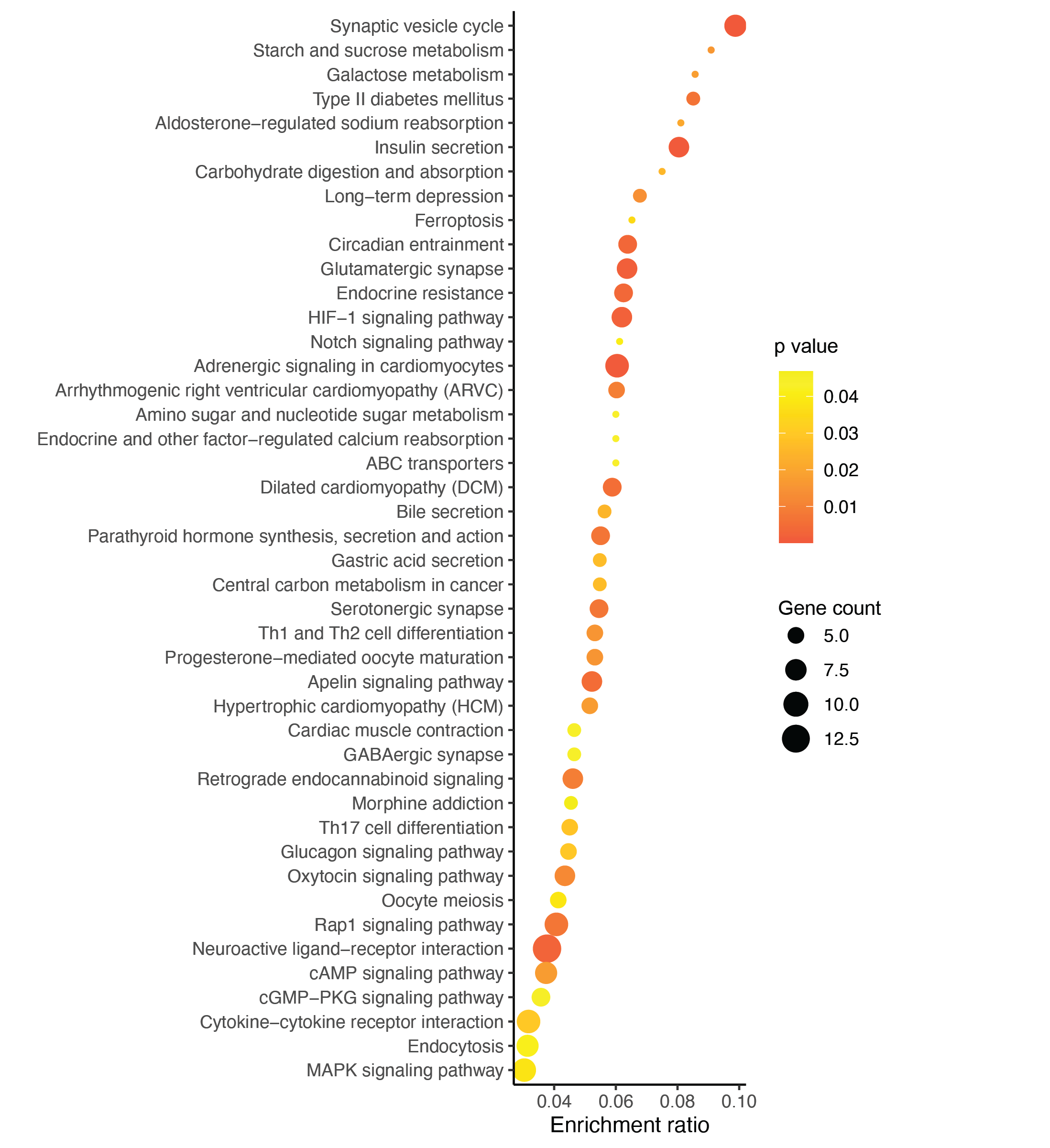


**Figure S10:** Gene regulatory pathways that are potentially activated due to MVA infection, as identified by Kegg Orthology analysis of upregulated genes. Pathways are shown in the decreasing order of their gene enrichment ratio. (Extended list of main text Figure 6a, right panel)


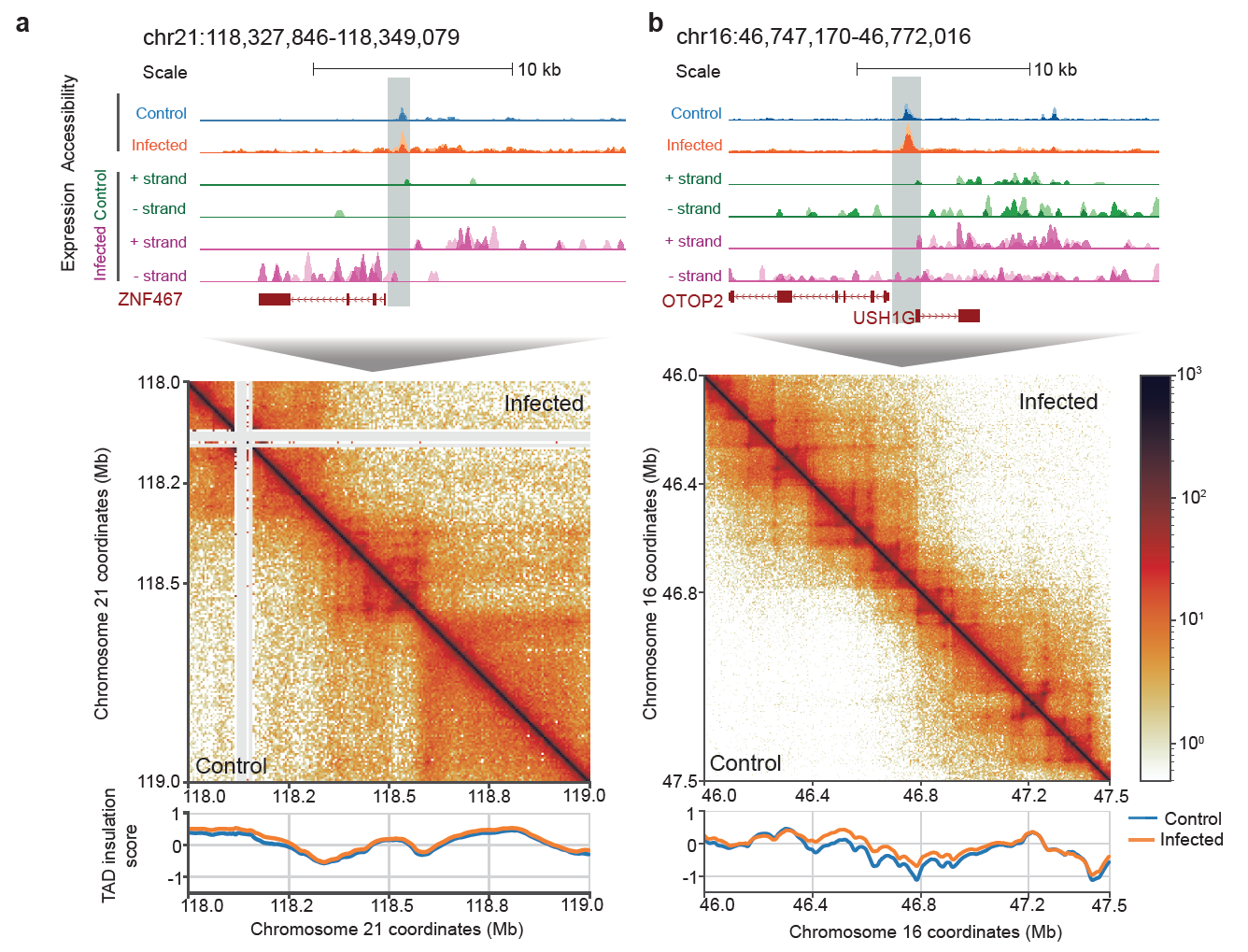


**Figure S11: a)**, **b)** ATAC-seq and gene expression signal tracks from 24 hpi at example regions (same regions as shown in the main text Figure 6a and 6b) with infection-biased accessibility and gene expression shown in the top panel. Replicates are overlaid. Blue: accessibility in mock-infected control cells. Orange: accessibility in MVA-infected cells. Green: control gene expression. Purple: infected gene expression. Corresponding Hi-C contact heat map of a larger region including the differential accessible window is shown in the middle panel. Control and MVA-infected contacts are shown in the lower and upper diagonal parts of the figure, respectively. TAD insulation score for the corresponding region is shown in the bottom panel. Control (blue) and infected (orange) TAD insulation scores are overlaid. While the region shown in a) displays overall higher TAD mixing with a weaker insulation score in both mock-infected control and infected samples, as evident from TAD insulation score plot, the region shown in b) possesses marginally increased TAD mixing in infected cells.


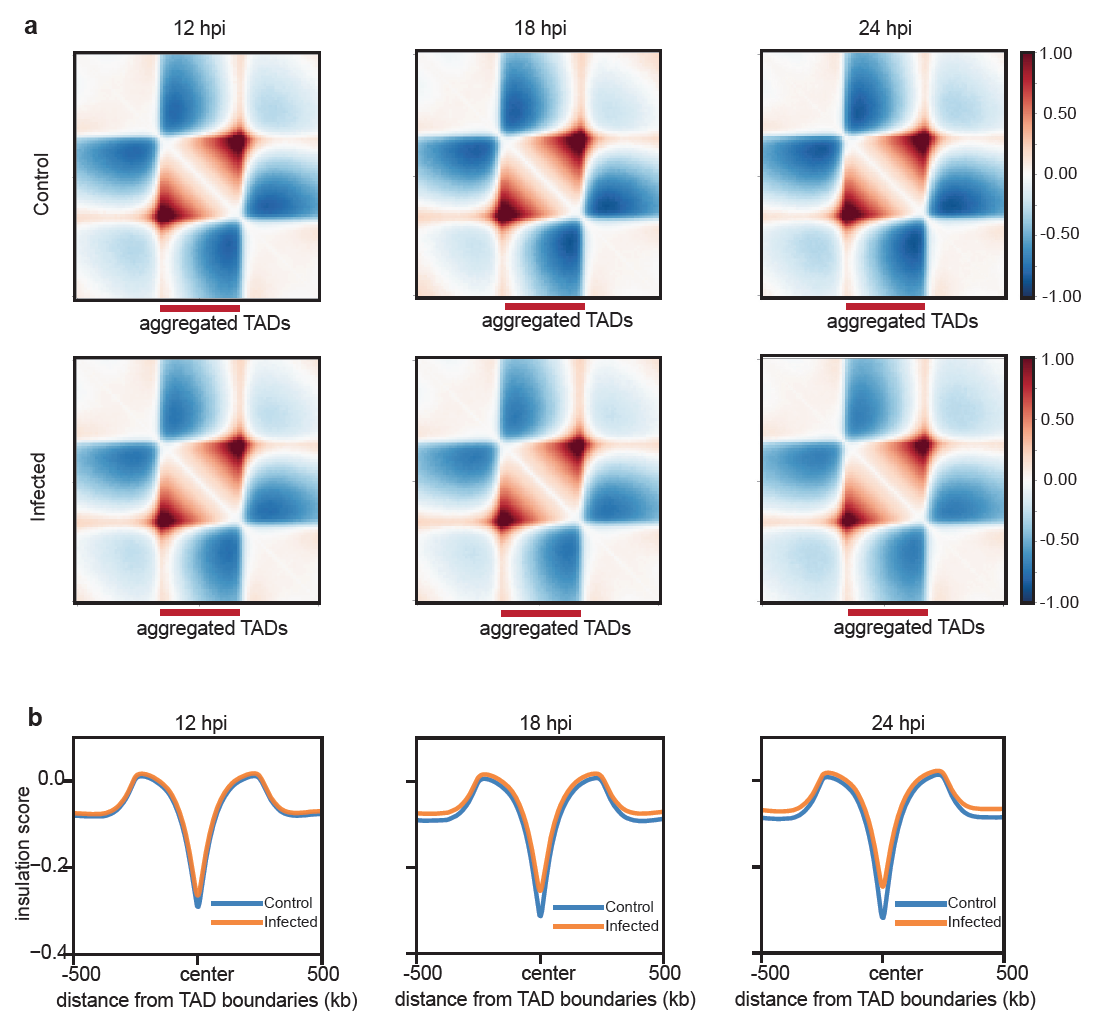


**Figure S12:** **a)** Mock-infected control (top) and MVA-infected (bottom) average log_2_ observed over expected contact frequency within scaled aggregated TADs at 12, 18 and 24 hpi are shown as heatmaps. TAD location is denoted by the maroon bar and the additional same sized flanking region is included on both sides. **b)** A profile plot of insulation scores (control: blue, infected: orange) computed from all TAD boundary midpoints ± 500 kb region identified at 12, 18, and 24 hpi respectively.

| **Biological Replicate** | **Sample** | **Total read-pairs** | **Hi-C contacts** | **Contacts**  **(Per condition)** | **Loops called** |
| --- | --- | --- | --- | --- | --- |
| 1 | 12hr Control | 559,483,569 | 256430862 | 783,687,719 | 10146 |
| 2 | 12hr Control | 886,507,172 | 534469766 |  | 17667 |
| 1 | 12hr Infected | 777,613,496 | 270746905 | 712,124,938 | 8771 |
| 2 | 12hr Infected | 770,418,243 | 463757732 |  | 15975 |
| 1 | 18hr Control | 761,567,093 | 327008067 | 702,267,757 | 13569 |
| 2 | 18hr Control | 610,190,915 | 381729147 |  | 17416 |
| 1 | 18hr Infected | 646,594,007 | 279263882 | 653,725,550 | 10064 |
| 2 | 18hr Infected | 655,739,644 | 408610556 |  | 15379 |
| 1 | 24hr Control | 725,697,285 | 449311017 | 846,643,183 | 17871 |
| 2 | 24hr Control | 619,039,813 | 405023378 |  | 17092 |
| 1 | 24hr Infected | 781,644,457 | 462522234 | 799,501,451 | 14718 |
| 2 | 24hr Infected | 607,026,371 | 387415043 |  | 13988 |

Table S1: Hi-C data summary.

| **Biological Replicate** | **Sample** | **Total read pairs** | **ATAC-seq peaks in assembled chromosomes** | **FRiP score** |
| --- | --- | --- | --- | --- |
| 1 | 12hr Control | 42,598,767 | 126,980 | 0.69 |
| 2 | 12hr Control | 22,250,266 | 148,912 | 0.51 |
| 1 | 12hr Infected | 39,289,957 | 98,872 | 0.47 |
| 2 | 12hr Infected | 65,728,373 | 126,769 | 0.54 |
| 1 | 18hr Control | 17,094,491 | 114,885 | 0.75 |
| 2 | 18hr Control | 64,577,918 | 135,385 | 0.61 |
| 1 | 18hr Infected | 29,811,600 | 57,763 | 0.46 |
| 2 | 18hr Infected | 58,849,770 | 75,849 | 0.38 |
| 1 | 24hr Control | 21,883,403 | 110,433 | 0.72 |
| 2 | 24hr Control | 78,668,288 | 162,898 | 0.59 |
| 1 | 24hr Infected | 35,202,941 | 835,69 | 0.38 |
| 2 | 24hr Infected | 86,633,067 | 140,331 | 0.43 |

Table S2: ATAC-seq data summary

Table S3: RNA-seq data summary

| **Biological Replicate** | **Sample** | **Total**  **read pairs** | **Total aligned read pairs** | **Genes with 5X or greater read depth** |
| --- | --- | --- | --- | --- |
| 1 | 12hr Control | 16,724,888 | 14,188,215 | 14264 |
| 2 | 12hr Control | 7,853,774 | 6,475,281 | 12081 |
| 1 | 12hr Infected | 40,441,286 | 34,712,486 | 17491 |
| 2 | 12hr Infected | 14,313,472 | 11,975,560 | 15458 |
| 1 | 18hr Control | 14,881,111 | 12,319,322 | 15178 |
| 2 | 18hr Control | 13,288,297 | 11,465,064 | 15179 |
| 1 | 18hr Infected | 9,962,273 | 8,520,981 | 14161 |
| 2 | 18hr Infected | 17,880,406 | 15,165,670 | 15599 |
| 1 | 24hr Control | 19,478,850 | 16,443,010 | 15774 |
| 2 | 24hr Control | 19,602,211 | 16,540,109 | 15879 |
| 1 | 24hr Infected | 17,689,469 | 14,446,357 | 15318 |
| 2 | 24hr Infected | 17,537,009 | 14,926,234 | 15283 |

| **Replicate** | **Sample** | **Loops identified with resolution** | | | **Total** |
| --- | --- | --- | --- | --- | --- |
|  |  | **5 kb** | **10 kb** | **25 kb** |  |
| 1 | 12 hr Control | 2888 | 3749 | 3509 | 10146 |
| 2 | 12 hr Control | 8653 | 5615 | 3399 | 17667 |
| 1 | 12 hr Infected | 2007 | 3183 | 3581 | 8771 |
| 2 | 12 hr Infected | 7173 | 5119 | 3683 | 15975 |
| 1 | 18 hr Control | 4897 | 4726 | 3946 | 13569 |
| 2 | 18 hr Control | 8127 | 5444 | 3845 | 17416 |
| 1 | 18 hr Infected | 2902 | 3497 | 3665 | 10064 |
| 2 | 18 hr Infected | 6705 | 4702 | 3972 | 15379 |
| 1 | 24 hr Control | 8672 | 5603 | 3596 | 17871 |
| 2 | 24 hr Control | 7954 | 5376 | 3762 | 17092 |
| 1 | 24 hr Infected | 6229 | 4803 | 3686 | 14718 |
| 2 | 24 hr Infected | 5679 | 4419 | 3890 | 13988 |

Table S4: Loop summary
