# Supplementary material for: Multi-omics analysis reveals the dynamic interplay between Vero host chromatin structure and function during vaccinia virus infection
Source: Commun Biol. 2024 Jun 11;7:721. doi: 10.1038/s42003-024-06389-x (PMC11166932; doi:10.1038/s42003-024-06389-x)
Supplement: Supplementary file 3 — Reporting Summary [file 42003_2024_6389_MOESM3_ESM.pdf]

nature portfolio

Corresponding author(s): Christina Rene Steadman

Last updated by author(s): 5/11/2024

Reporting Summary

Nature Portfolio wishes to improve the reproducibility of the work that we publish. This form provides structure for consistency and transparency in reporting. For further information on Nature Portfolio policies, see our Editorial Policies and the Editorial Policy Checklist.

Statistics

For all statistical analyses, confirm that the following items are present in the figure legend, table legend, main text, or Methods section.

n/a	Confirmed	
☐	☒	The exact sample size (*n*) for each experimental group/condition, given as a discrete number and unit of measurement
☐	☒	A statement on whether measurements were taken from distinct samples or whether the same sample was measured repeatedly
☐	☒	The statistical test(s) used AND whether they are one- or two-sided<br>*Only common tests should be described solely by name; describe more complex techniques in the Methods section.*
☐	☒	A description of all covariates tested
☐	☒	A description of any assumptions or corrections, such as tests of normality and adjustment for multiple comparisons
☐	☒	A full description of the statistical parameters including central tendency (e.g. means) or other basic estimates (e.g. regression coefficient) AND variation (e.g. standard deviation) or associated estimates of uncertainty (e.g. confidence intervals)
☐	☒	For null hypothesis testing, the test statistic (e.g. *F*, *t*, *r*) with confidence intervals, effect sizes, degrees of freedom and *P* value noted<br>*Give P values as exact values whenever suitable.*
☐	☒	For Bayesian analysis, information on the choice of priors and Markov chain Monte Carlo settings
☐	☒	For hierarchical and complex designs, identification of the appropriate level for tests and full reporting of outcomes
☐	☒	Estimates of effect sizes (e.g. Cohen's *d*, Pearson's *r*), indicating how they were calculated

Our web collection on statistics for biologists contains articles on many of the points above.

Software and code

Policy information about availability of computer code

Data collection	No software was used for data collection
Data analysis	BWA v0.7.17, fastp v0.23.4, macs2 v2.2.7.1, samtools v1.16.1, deeptools v3.5.3, bedtools v2.30.0, DESeq2 v1.38.0, R v4.2.0, ggplot2 v3.5.0, Juicer v1.6.0, juicer_tools_1.22.01, fans v0.9.26, multiHiCcompare v1.16.0, KOBAS 2.0, AGAT v1.2.0, FastQC v0.11.9, SeqMonk v1.48.1, STAR 2.4.10b, Htseq v2.0.4, bio v1.5.9, ImageJ v. 1.53

For manuscripts utilizing custom algorithms or software that are central to the research but not yet described in published literature, software must be made available to editors and reviewers. We strongly encourage code deposition in a community repository (e.g. GitHub). See the Nature Portfolio guidelines for submitting code & software for further information.

Data

Policy information about availability of data

All manuscripts must include a data availability statement. This statement should provide the following information, where applicable:

- Accession codes, unique identifiers, or web links for publicly available datasets
- A description of any restrictions on data availability
- For clinical datasets or third party data, please ensure that the statement adheres to our policy

All sequencing raw data generated during this study from ATAC-seq, RNA-seq and Hi-C experiments are available in the SRA repository, under the BioProject

accession number PRJNA1037174. Processed data files supporting the the conclusions of this article are available in GEO repository with accession number: GSE248052.

Research involving human participants, their data, or biological material

Policy information about studies with human participants or human data. See also policy information about sex, gender (identity/presentation), and sexual orientation and race, ethnicity and racism.

Reporting on sex and gender	NA
Reporting on race, ethnicity, or other socially relevant groupings	NA
Population characteristics	NA
Recruitment	NA
Ethics oversight	NA

Note that full information on the approval of the study protocol must also be provided in the manuscript.

Field-specific reporting

Please select the one below that is the best fit for your research. If you are not sure, read the appropriate sections before making your selection.

☒ Life sciences ☐ Behavioural & social sciences ☐ Ecological, evolutionary & environmental sciences

For a reference copy of the document with all sections, see nature.com/documents/nr-reporting-summary-flat.pdf

Life sciences study design

All studies must disclose on these points even when the disclosure is negative.

Sample size	12 Hi-C, 12 ATAc-seq, 12 RNA-seq data sets
Data exclusions	No samples were excluded
Replication	Two biological replicates per condition were used for for all assays. All statistical tests except TAD strength analysis were performed on replicate separate data sets and differential features were identified as the ones supported by p-value <0.05. For maximum signal strength, replicate combined Hi-C maps were used for TAD strength analysis.
Randomization	Each biological replicate was treated independently and cells were divided into assays randomly. For fair comparison, time matched control samples were processed in the same manner.
Blinding	Culture collection was numbered as were libraries; alignment and calling was performed on numbered samples. Metadata was revealed for comparative analysis between conditions (time and infection).

Reporting for specific materials, systems and methods

We require information from authors about some types of materials, experimental systems and methods used in many studies. Here, indicate whether each material, system or method listed is relevant to your study. If you are not sure if a list item applies to your research, read the appropriate section before selecting a response.

Materials & experimental systems

n/a	Involved in the study	
☐	☒	Antibodies
☐	☒	Eukaryotic cell lines
☒	☐	Palaeontology and archaeology
☒	☐	Animals and other organisms
☒	☐	Clinical data
☒	☐	Dual use research of concern
☒	☐	Plants

Methods

n/a	Involved in the study	
☒	☐	ChIP-seq
☒	☐	Flow cytometry
☒	☐	MRI-based neuroimaging

Antibodies

Antibodies used	Vaccinia Virus Antibody (8115), Santa Cruz, Cat # sc-58210; rabbit anti mouse IgG-FITC, sc-358916
Validation	Antibodies were validated by manufacturer.

Eukaryotic cell lines

Policy information about cell lines and Sex and Gender in Research

Cell line source(s)	Cryopreserved Vero cells, derived from the kidney tissue of a normal adult African green monkey, were sourced from the ATCC (Cat # CCL-81).
Authentication	Not authenticated
Mycoplasma contamination	Not tested for mycoplasma contamination
Commonly misidentified lines (See ICLAC register)	Vero is not one of the misidentified cell lines per the ICLAC register.

Plants

Seed stocks	NA
Novel plant genotypes	NA
Authentication	NA
